# Field-friendly serological tests for determination of *M. leprae*-specific antibodies

**DOI:** 10.1038/s41598-017-07803-7

**Published:** 2017-08-21

**Authors:** Anouk van Hooij, Elisa M. Tjon Kon Fat, Susan J. F. van den Eeden, Louis Wilson, Moises Batista da Silva, Claudio G. Salgado, John S. Spencer, Paul L. A. M. Corstjens, Annemieke Geluk

**Affiliations:** 1 Dept. of Infectious Diseases, Leiden University Medical Center, Leiden, The Netherlands; 2 Dept. Molecular Cell Biology and Dept. Reumatology, Leiden University Medical Center, Leiden, The Netherlands; 3 Dept. of Microbiology, Immunology and Pathology, Colorado State University, Fort Collins, USA; 4 Laboratório de Dermato-Imunologia, Instituto de Ciências Biológicas, Universidade Federal do Pará, Marituba, Pará Brazil

## Abstract

Early detection of leprosy is key to reduce the ongoing transmission. Antibodies directed against *M. leprae* PGL-I represent a useful biomarker for detecting multibacillary (MB) patients. Since efficient leprosy diagnosis requires field-friendly test conditions, we evaluated two rapid lateral flow assays (LFA) for detection of *Mycobacterium leprae*-specific antibodies: the visual immunogold OnSite Leprosy Ab Rapid test [Gold-LFA] and the quantitative, luminescent up-converting phosphor anti-PGL-I test [UCP-LFA]. Test performance was assessed in independent cohorts originating from three endemic areas. In the Philippine cohort comprising patients with high bacillary indices (BI; average:4,9), 94%(n = 161) of MB patients were identified by UCP-LFA and 78%(n = 133) by Gold-LFA. In the Bangladeshi cohort, including mainly MB patients with low BI (average:1), 41%(n = 14) and 44%(n = 15) were detected by UCP-LFA and Gold-LFA, respectively. In the third cohort of schoolchildren from a leprosy hyperendemic region in Brazil, both tests detected 28%(n = 17) seropositivity. Both rapid tests corresponded well with BI(p < 0.0001), with a fairly higher sensitivity obtained with the UCP-LFA assay. However, due to the spectral character of leprosy, additional, cellular biomarkers are required to detect patients with low BIs. Therefore, the UCP-LFA platform, which allows multiplexing with differential biomarkers, offers more cutting-edge potential for diagnosis across the whole leprosy spectrum.

## Introduction

Leprosy, an infectious disease caused by *Mycobacterium leprae* (*M. leprae*), still poses a major health threat in developing countries. The availability of effective multi-drug therapy (MDT) has decreased the global disease burden significantly, however, the annual new case detection rate has remained virtually stable during the past decade which undeniably points towards the continuation of bacterial transmission. Mis- or delayed diagnosis frequently occurs as leprosy diagnosis still relies on clinical symptoms^1^. These symptoms typically take 2–6 years, but also up to 20 years, to become manifest^2^. Moreover, as the majority of people have sufficient natural immunity to (myco)bacterial infection and will not progress to disease^3^, a small proportion (1–5%) of *M. leprae* infected individuals will actually develop clinical symptoms. During subclinical *M. leprae* infection the host, without being aware of the infectious state, may transmit the bacteria, allowing transmission to continue, especially among close contacts of the infected individuals^4, 5^. Diagnostic tests for early detection of leprosy, allowing adequate treatment of early-stage leprosy and *M. leprae* infection, could therefore make significant differences in transmission and clinical outcomes.

Leprosy presents as a spectral disease, ranging from a dominant cellular phenotype with the ability to mount a cellular response that leads to effective killing of *M. leprae*, to an immune response characterized mostly by humoral immunity against *M. leprae* ^6^. Within the leprosy spectrum five disease types can be identified according to the Ridley Joplin classification^7^: tuberculoid (TT), borderline tuberculoid (BT), borderline (BB), borderline lepromatous (BL) and lepromatous leprosy (LL). Alternatively, the WHO classification is based on the number of skin lesions and nerve involvement and classifies leprosy as multibacillary (MB; > 5 lesions) or paucibacillary (PB; ≤ 5 lesions)^2^ in which PB is predominantly associated with the cellular phenotype and MB with the humoral immune response.

A useful biomarker for leprosy, predominantly for MB patients, is the level of IgM antibodies directed against the *M. leprae*-specific phenolic glycolipid I (PGL-I)^8, 9^. Moreover, upon effective treatment of leprosy IgM levels drop and can therefore be used to monitor efficacy of leprosy treatment^10^.

Leprosy endemic areas are often short of sophisticated laboratories which makes it imperative to develop diagnostic tests for the detection of PGL-I antibodies suitable for field settings. The aim of this study was to evaluate two recently designed field-friendly lateral flow assays (LFAs), the OnSite Leprosy Ab Rapid test^11^ and the in-house developed PGL-I up-converting phosphor (UCP)-LFA^12^.

The OnSite Leprosy Ab Rapid test is an immunochromatographic LFA, detecting IgM antibodies against PGL-I and IgG antibodies to LID-1. The latter fusion protein, encoded by the genes for ML0405 and ML2331, has been shown to be a useful diagnostic marker for MB leprosy^13, 14^.

UCP-LFAs have previously demonstrated applicability for detection and monitoring of a variety of analytes^15–17^, including cellular biomarkers for *Mycobacterium tuberculosis* ^18, 19^. Therefore, we developed user-friendly UCP-LFAs for the detection of IgM antibodies against PGL-I, which proved robust in multiple (field) studies^10, 12, 20^. The luminescent UCP-label enables quantitative determination of IgM levels using portable readers, whereas the colloidal gold label of the OnSite Leprosy Ab Rapid test (Gold-LFA) generates qualitative results which are visually inspected. Test performance of these two different field-friendly LFA formats was assessed using identical sera of 3 cohorts derived from different leprosy endemic areas in the Philippines, Bangladesh and Brazil.

## Results

### Performance of UCP-LFA and Gold-LFA in selected, polar leprosy patients

**Figure 1:**
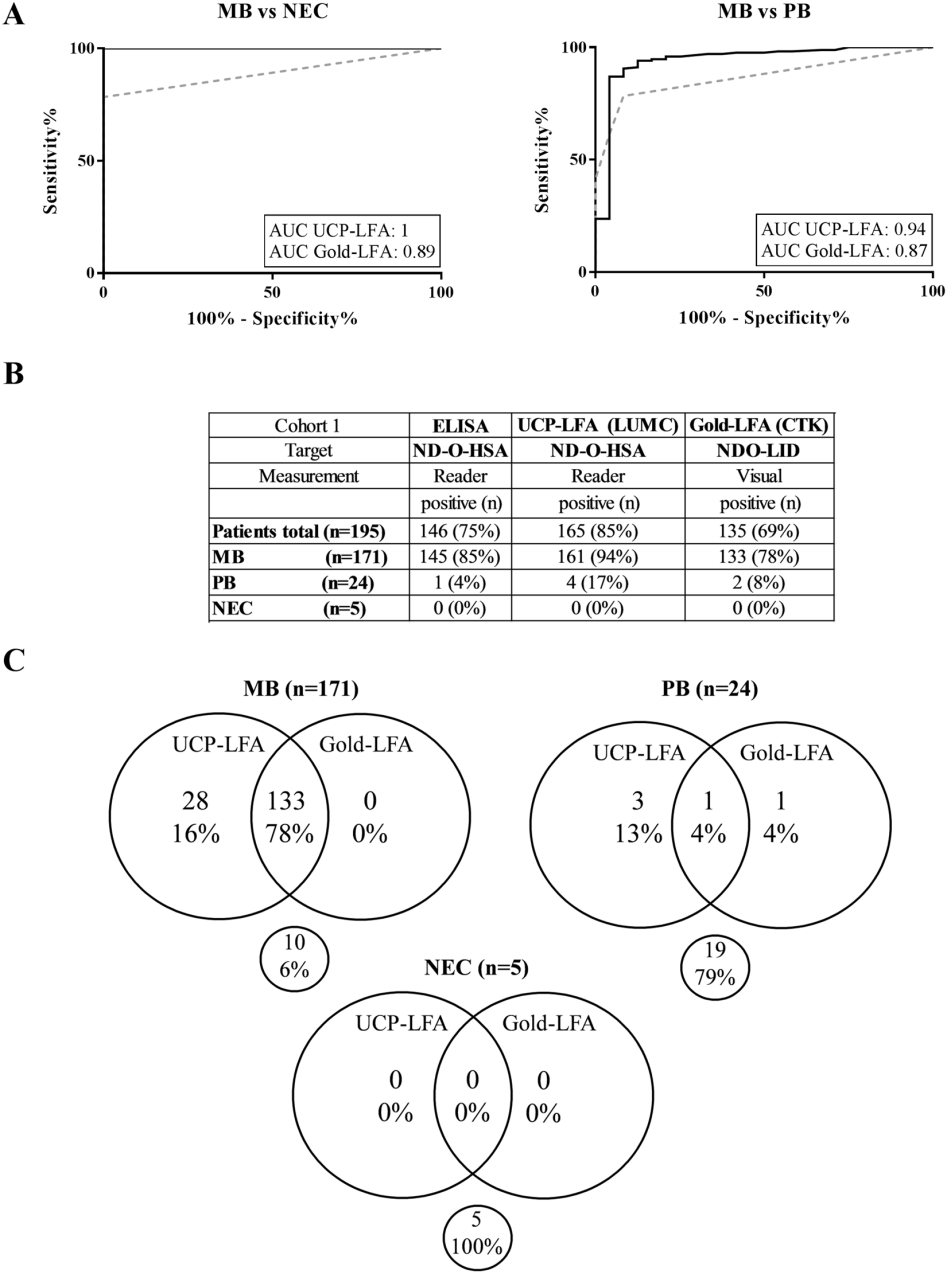
Test performance of UCP-LFA and Gold-LFA in Philippine leprosy patients. Test performance was assessed using serum of 171 multibacillary (MB) and 24 paucibacillary (PB) patients. Non-endemic controls were included as negative control. (**A**) Receiver operating characteristics (ROC) curves showing the distinction between MB patients and NEC (left panel) and MB and PB patients (right panel) for the UCP-LFA (solid line) and Gold-LFA (dotted line). Areas under curve (AUCs) are displayed for both tests. (**B**) Number and percentage of positive individuals per test (ELISA, UCP-LFA, Gold-LFA) are shown for each test group. (**C**) Venn diagrams showing the concordance in positive individuals between UCP-LFA and Gold-LFA per test group.

To analyze the performance of the UCP-LFA and the Gold-LFA, we first analyzed a selection of Philippine polar leprosy patients (cohort 1, n = 200). MB patients (n = 171) could be adequately distinguished from nonendemic controls (NEC; n = 5) using the UCP-LFA (AUC:1; cut-off:0.29, sensitivity: 94%, specificity: 100%) or the Gold-LFA (AUC:0.89; cut-off:0.5, sensitivity: 78%, specificity: 100%) (Fig. 1A). In the PGL-I ELISA, 145 out of 171 MB patients (85%) were positive (Fig. 1B). The UCP-LFA identified 161 (94%) MB patients as positive, whereas for 133 (78%) MB patients a positive test band was observed in the Gold-LFA. Furthermore, all 5 NEC included as negative controls were confirmed negative in both Gold-LFA and UCP-LFA.

Of the 24 PB patients, one (4%) was positive in the PGL-I ELISA, 2 (8%) in the Gold-LFA and 4 (17%) in the UCP-LFA (Fig. 1B). With respect to disease classification both tests distinguished MB and PB patients very well, providing an AUC of 0.94 for the UCP-LFA (sensitivity: 94%; specificity: 81%) and 0.87 for the Gold-LFA (sensitivity: 78%; specificity: 94%) (Fig. 1A).

Assessment of test concordance showed that the majority (n = 133; 78%) of MB patients were detected by both LFAs, whereas 28 (16%) were identified by UCP-LFA only (Fig. 1C; Supplementary Figure S2). Of these 28 individuals, 22 (79%) showed ELISA values around the cut-off (0.1–0.5), indicating sensitive detection of low PGL-I positives. Out of 24 PB patients one was positive in both tests, one in the Gold-LFA only, 3 in the UCP-LFA only and 19 were negative in both tests.

In summary, although some samples showed discordant results between the 2 tests (Supplementary Figure S2) in the Philippine cohort both LF-based tests identified the majority of MB patients (≥78%) and could distinguish MB patients from PB patients or NEC.

### Test performance of UCP-LFA and Gold-LFA in a Bangladeshi cohort

**Figure 2:**
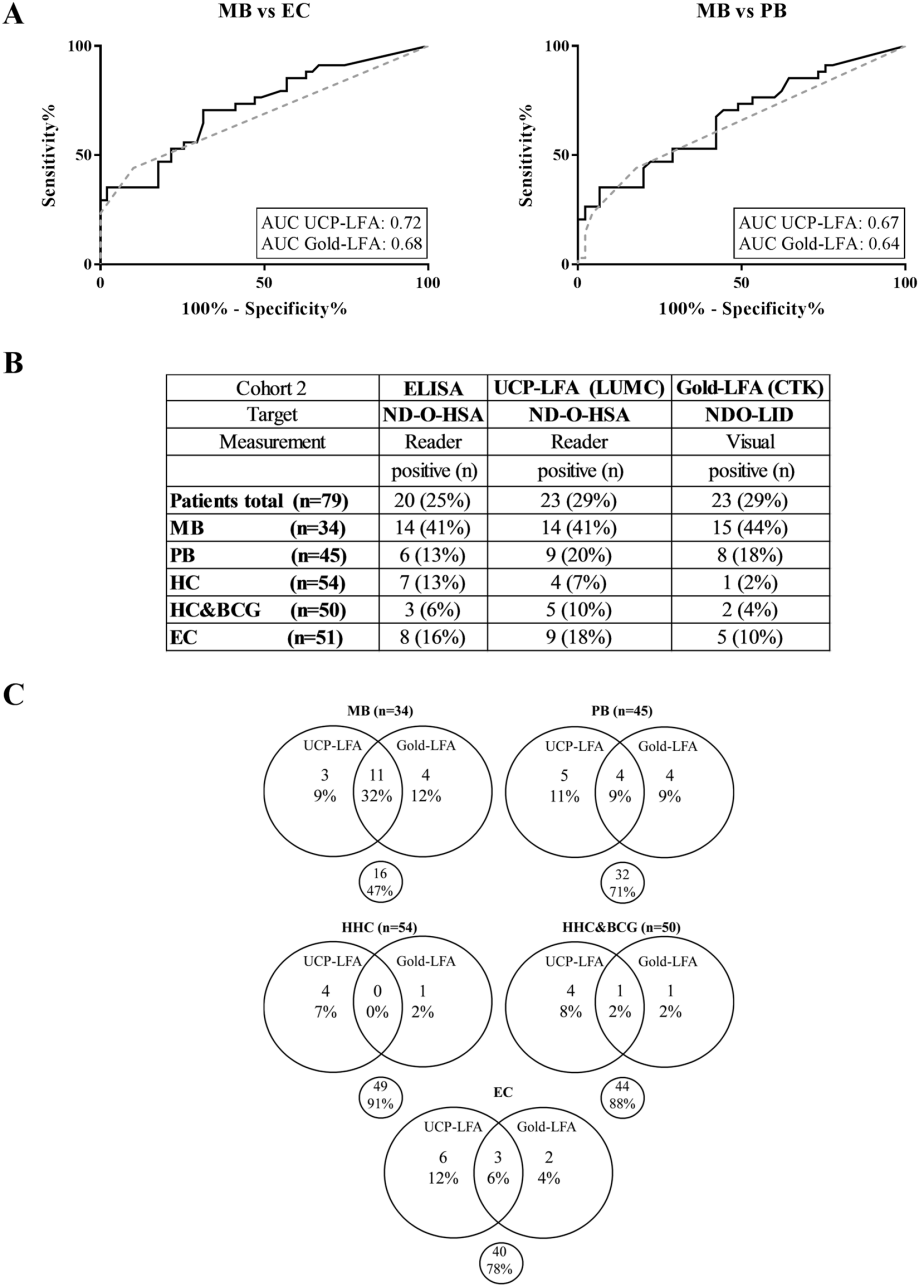
Test performance of UCP-LFA and Gold-LFA in a Bangladeshi cohort. Test performance was assessed using serum of 34 multibacillary (MB), 45 paucibacillary (PB) patients, 104 healthy household contacts (BGC-vaccinated [n = 50], non-vaccinated [n = 54]) and 51 endemic controls (EC). (**A**) Receiver operating characteristics (ROC) curves showing the distinction between MB patients and EC (left panel) and MB and PB patients (right panel) for the UCP-LFA (solid line) and Gold-LFA (dotted line). Areas under curve (AUCs) are displayed for both tests. (**B**) Number and percentage of positive individuals per test (ELISA, UCP-LFA, Gold-LFA) are shown for each test group. (**C**) Venn diagrams showing the concordance in positive individuals between UCP-LFA and Gold-LFA per test group.

Since both LFAs performed well in cohort 1, which was selected to include polar types of leprosy, we next evaluated test performance in an unbiased population characterized by representation of less polar forms of leprosy and MB patients with low BIs, consisting of leprosy patients, healthy household contacts (HHC) and endemic controls (EC) from Bangladesh (Cohort 2). For EC (n = 51), PGL-I seropositivity was detected, 8 (16%) in the PGL-I ELISA, 9 (18%) in UCP-LFA and 5 (10%) in the Gold-LFA, resulting in AUCs for MB diagnosis of 0.72 and 0.68 for the PGL-I UCP-LFA and Gold-LFA, respectively (Fig. 2A,B). For classification of leprosy into MB or PB, AUCs were comparable, 0.67 for the UCP-LFA and 0.64 for the Gold-LFA (Fig. 2A).

14 out of 34 MB patients (41%), the majority of whom had a low or negative BI (Supplementary Figure S1), were positive in the PGL-I ELISA, which was similarly reflected in the UCP-LFA (n = 14; 41%) and the Gold-LFA (n = 15; 44%) (Fig. 2B). As in the PGL-I ELISA, seropositivity was also observed in both HHC groups (with or without BCG vaccination). Besides only a low number in all test groups in cohort 2 being seropositive in either of the 2 tests, the PGL-I UCP-LFA and Gold-LFA also detected different seropositive HHC (8 seropositive in UCP-LFA only and 2 in Gold-LFA only; Fig. 2C; Supplementary Figure S1), indicating that detection of different antibodies in these two test formats can identify different individuals. In patients, 7 MB and 9 PB patients were seropositive in either the UCP-LFA or Gold-LFA, of whom 75% (n = 12) showed a weakly positive test response (Supplementary Figure S2, Fig. 2C).

### Application of UCP-LFA and Gold-LFA in a hyperendemic region in Brazil

Samples of cohort 3 (n = 60) were collected in a region considered hyperendemic for leprosy in Brazil. Although none of the individuals showed clinical signs of leprosy, both the UCP-LFA and Gold-LFA detected antibodies to *M. leprae* specific antigens in 17 individuals (28%), whereas the conventional ELISA detected antibodies in 11 individuals (18%). All individuals seropositive by ELISA were also detected by the UCP-LFA and Gold-LFA. Moreover, the majority of individuals detected by UCP-LFA and Gold-LFA showed concordant results (n = 15), 2 individuals were detected by UCP-LFA only and 2 others by Gold -LFA only (supplementary Figure S1) indicating similar test performance for the 2 field-friendly assays.

Moreover, we evaluated both test in TB patients, which were all negative showing the lack of cross-reactivity at antibody level with other mycobacterial infections (Supplementary Table S1).

### Correlation of lateral flow tests with Bacterial Index

Antibodies against PGL-I and LID-1 are reported to predominantly identify MB patients. Consistent with this the majority of MB patients was identified in cohort 1 (MB patients with high bacterial index (BI; average: 4,9; range: 3,2–6)), whereas in cohort 2 (MB patients with a low or negative BI (average: 1; range:0–5)) more than half of the MB patients was not seropositive in either of the tests (UCP-LFA:56%; Gold-LFA:59%).

**Figure 3:**
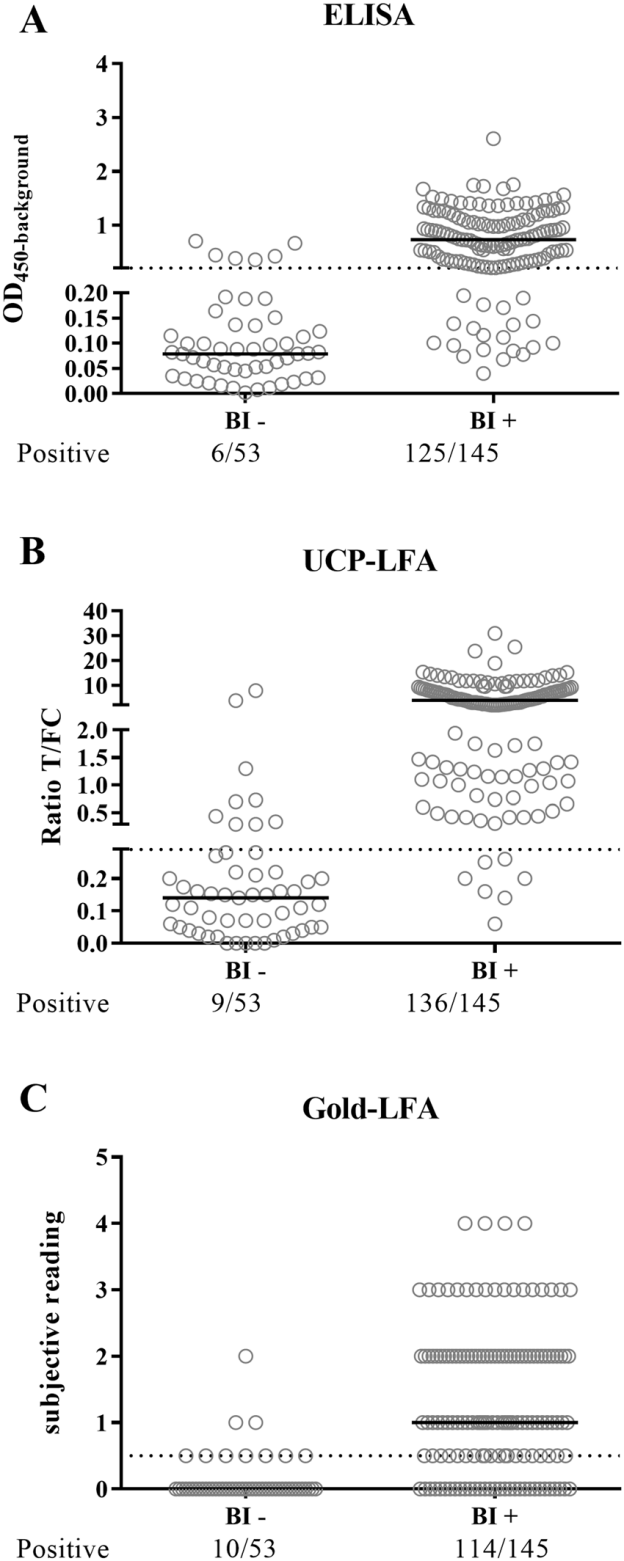
Test correspondence with bacterial index. Leprosy patients of which the BI was assessed were stratified by bacterial index (BI negative [n = 53] and positive [n = 145]) to evaluate the correlation between BI and LF test results. (**A**) ELISA data stratified by BI, the cut-off of OD_450_-background (=0.2) is visualized by the dotted line. 125 BI^+^ patients and 6 BI^−^ patients showed a positive result in ELISA. Values significantly differed between BI^−^ and BI^+^ patients (p < 0.0001) (**B**) UCP-LFA data stratified by BI, the positive cut-off of ratio 0.29 is visualized by the dotted line. 136 BI^+^ patients and 9 BI^−^ negative patients showed a positive result in the UCP-LFA. Values significantly differed between BI^−^ and BI^+^ patients (p < 0.0001) (**C**) Gold-LFA data stratified by BI, the cut-off for a positively scored samples (observed test band > 0.5) is visualized by the dotted line. 114 BI^+^ patients and 10 BI^−^ patients were scored as positive in the Gold-LFA. Values significantly differed between BI^−^and BI^+^ patients (p < 0.0001).

To examine the correlation between the test results and BI, leprosy patients from both cohorts (n = 198) were stratified by BI (BI^+^ [n = 145], BI^−^[n = 53]). In ELISA, 125 (86%) of BI^+^ patients showed a positive anti-PGL-I titer, in the UCP-LFA 136 (94%) and in the Gold-LFA 114 (78%) (Fig. 3). Of the 53 BI^−^ patients 6 (11%) showed a positive anti-PGL-I titer in ELISA, 9 (17%) in the UCP-LFA and 10 (19%) in the Gold-LFA. In all tests, significantly higher positive values were observed in the BI positive group (p < 0.0001), indicating strong correlation with BI and implying the need of other (cellular) biomarkers to diagnose patients with low BI.

## Discussion

User-friendly tools to facilitate the diagnosis of leprosy or/*M. leprae* infection are urgently needed to tackle the ongoing transmission. In this study we have compared the UCP-LFA and the OnSite Leprosy Ab Rapid test (Gold-LFA), two user-friendly tools for the identification of *M. leprae* specific humoral immune responses. The UCP-LFA detects IgM antibodies directed against PGL-I, whereas the Gold-LFA additionally identifies IgG antibodies directed against LID-1. In a head-to-head comparison of these two LFA tests, the UCP-LFA identified similar (cohort 2 [41%] and 3 [28%]) or more (cohort 1 [94%]) MB patients than the Gold-LFA (cohort 1 [78%], cohort 2 [44%], cohort 3 [28%]). Especially in MB patients with a positive BI the UCP-LFA turned out to be more sensitive than the Gold-LFA, identifying 94% of these patients and outperforming the conventional PGL-I ELISA. Despite the similarity in test results between both tests, a number of individuals were detected exclusively by either the UCP-LFA (n = 55) or the Gold-LFA (n = 15). On one hand, this discrepancy can be explained by the more sensitive quantitative detection of anti-PGL-I IgM in the UCP-LFA due to the use of a luminescence label in contrast to a visual, gold label^21^. On the other hand, though less frequently occurring, seropositivity due to the presence of LID-1-specific anti-IgG may detect other samples only in the Gold-LFA. Furthermore, since leprosy patients may present only anti-PGL-I IgG and not IgM^22^ the inclusion of detection of IgG specific for PGL-I in diagnostic tests for leprosy should also be considered.

An essential difference between the two antibody tests is the interpretation of test results, whereas the UCP-LFA results are objectively measured by a calibrated reader, the Gold-LFA test relies on somewhat more subjective visual evaluation by operators. Especially in the field operator differences should be taken into account, and re-examination by a second individual is therefore vital. This should be done within 10–15 minutes after the test is performed^11^ to acquire reliable results. In contrast, the UCP-LFA strips can be permanently stored and sent to a reference lab for re-analysis.

The use of a reader (Smart Reader) to analyse immunogold LF strips similar to the Gold-LFA test for detection of *M. leprae*-specific antibodies has been described^23, 24^ and demonstrated that results by visual interpretation and results read by Smart Reader agreed moderately (κ index: 0,55)^25^. However, haemolysis of red blood cells when using the immunogold label may hamper accurate measurements and visualisation of low positives, contrary to the UCP-LFA format which is a virtually background-free reporter technology^21^.

In Bangladesh the majority of leprosy patients develop PB leprosy^2^, which were mostly negative in both LF tests used in this study as well as the standard ELISA for detection of antibodies against PGL-I. Moreover, PB patients cannot be distinguished from HHC or EC due to the seropositivity observed in these groups. Therefore, to identify not only the BI^+^ MB patients and increase specificity it is imperative to include detection of cellular markers in field friendly diagnostic assays for leprosy. Incorporation of the cellular markers IP-10, CCL4 and IL-10 into the same UCP-LFA format improved the assay sensitivity for detection of MB as well as PB patients in Bangladesh^20^.

The ability to simultaneously detect both humoral and cellular biomarkers of *M. leprae* infection in a single test, as proved feasible for the UCP-LF format^10^, can cover the entire spectrum of leprosy, therefore enabling more comprehensive diagnosis of leprosy patients.

## Materials and Methods

### Study participants

#### Cohort 1

Leprosy patients (LL/BL (n = 127), BT/TT (n = 24) and LL patients (n = 44; longitudinal samples of 9 patients), were diagnosed at the Cebu Skin Clinic and Leonard Wood Memorial (LWM) Center for Leprosy Research, Cebu, Philippines based on histological findings and clinical observations determined by experienced leprologists and a leprosy pathologist as previously described^8, 13^. Patients were categorized according to Ridley-Jopling classification^7^ and bacterial indices (BI) were determined. For some patients, serum was also obtained at specified intervals during treatment (at 1, 2, 3, 6, 9 and 12 months), and up to 2 years after the start of treatment^13^. Samples were collected from March 2007 until February 2012. All leprosy patient sera were collected at initial diagnosis prior to multidrug therapy (MDT). As a control group, non-BCG-vaccinated, U.S.-born healthy individuals with no known exposure to either tuberculosis or leprosy were included and designated as nonendemic controls (NEC; n = 5).

#### Cohort 2

Participants were recruited on a voluntary basis between January 2013 and December 2014 in leprosy endemic areas in Bangladesh as described previously^26^. Leprosy was diagnosed based on clinical, bacteriological and histological observations as previously described^27^. Clinical and demographic data were collected in a dedicated database. Participants were classified into five test groups: MB patients (n = 34; BL/LL = 8, BT = 26), PB patients (n = 45; BT = 41, TT = 4), healthy household contacts (HHC; n = 54) and BCG vaccinated HHC (HHC&BCG; n = 50) selected as previously described^20^. Control individuals without clinical disease symptoms from the same leprosy endemic area (endemic controls, EC; n = 51) were examined for the absence of clinical signs and symptoms of leprosy and tuberculosis. Staff of leprosy- or TB clinics were excluded as EC.

#### Cohort 3

Serum samples of schoolchildren were collected in the state of Pará, Brazil (n = 60), considered hyperendemic for leprosy (new case detection >4.0 per 10,000 population) as previously described^28^. Serum samples from all sources were coded to protect donor identities and were obtained with informed consent and/or with permission from the respective institutional review boards of each country.

#### Synthetic PGL-I

The disaccharide epitope (3,6-di-*O*-methyl-β-D-glucopyranosyl(1→4)2,3-di-*O*-methylrhamnopyranoside) of *M. leprae* specific native PGL-I glycolipid was synthesized and coupled to human serum albumin (synthetic PGL-I; designated ND-O-HSA, approximately 40 disaccharides per molecule)^19^. It was obtained through the Biodefense and Emerging Infections Research Resources Repository (http://www.beiresources.org/TBVTRMResearchMaterials/tabid/1431/Default.aspx).

#### PGL-I ELISA

Antibodies (IgM, IgG, IgA) against *M. leprae* PGL-I were detected by ELISA. Briefly, 200 ng ND-O-HSA was coated per well in 50 µl in 0.1 M Na_2_CO_3_/NaHCO_3_ buffer (pH 9.6) at 4 °C overnight. After blocking with 200 µl PBS/1%BSA/0.05% Tween 80 per well for 1 hour, 50 µl of 1:400 diluted sample was added and incubated for 2 hours at room temperature. Then, 50 µl of a 1:8000 dilution of anti-human IgG/IgM/IgA-HRP, (Dako P0212) in 0.05%Tween 20/PBS was incubated for 2 hours. In between each step the wells were washed 3 times with PBS/0,05% Tween 20. 50 µl of 3,3′,5,5′-Tetramethylbenzidine (TMB) was added and the color reaction was stopped using H_2_SO_4_ after 10–15 minutes. Absorbance was determined at a wavelength of 450 nm. Samples with an optical density at 450 nm (OD_450_) after correction for background OD (0,1%BSA in coating buffer) above 0.200 were considered positive. This threshold was determined by a threefold multiplication of an average NEC value.

#### The OnSite Leprosy Ab Rapid test

The OnSite Leprosy Ab Rapid test^11^ was purchased from CTK Biotech (San Diego, CA) and used according to the manufacturer’s instructions. Test band intensity was independently scored ranging from 0.5 to 4 by two independent individuals, in case of differences between operators the highest rated score was used^11^. The OnSite Leprosy Ab Rapid test is referred to as the Gold-LFA in this study.

#### PGL-I UCP-LFA

Anti-PGL-I IgM antibodies were detected as previously described^20^ using 100-fold diluted serum and an IgM-specific UCP conjugate (UCP^αIgM^). LF strips were scanned in a Packard FluoroCount microtiterplate reader adapted for measurement of the UCP label (980 nm IR excitation, 550 nm emission). Results are displayed as the ratio value between Test and Flow-Control signal based on relative fluorescence units (RFUs) measured at the respective lines. A threshold for positivity of 0.29 was determined by computing receiver operating characteristic (ROC) curves^20^.

#### Ethics

This study was performed according to the Helsinki Declaration as described previously^26^. The national Research Ethics Committee has approved the study protocol (Ref no. BMRC/NREC/2010–2013/1534 (Bangladesh) and Ethical Appreciation Certificate N° 26765414.0.0000.0018 (Brazil)). Participants were informed about the study-objectives, the samples and their right to refuse to take part or withdraw from the study without consequences for their treatment. Written informed consent was obtained before enrolment. Serum samples from the Philippines and NEC serum samples are part of a pre-existing collection at Colorado State University (JSS), and thus considered exempt from human subjects research. All patients received treatment according to national guidelines.

### Statistical analysis

Graphpad Prism version 7.00 for Windows (GraphPad Software, San Diego CA, USA) was used to perform Mann-Whitney U tests, plot ROC curves and calculate the area under curve (AUC).

## Electronic supplementary material

Supplementary information
