# Supplementary material for: Field-friendly serological tests for determination of M. leprae-specific antibodies
Source: Sci Rep. 2017 Aug 21;7:8868. doi: 10.1038/s41598-017-07803-7 (PMC5566372; doi:10.1038/s41598-017-07803-7)
Supplement: Supplementary file 1 — Supplementary information [file 41598_2017_7803_MOESM1_ESM.pdf]

SUPPLEMENTARY INFORMATION

FIELD-FRIENDLY SEROLOGICAL TESTS FOR DETERMINATION OF *M. LEPRAE*-SPECIFIC ANTIBODIES

Anouk van Hooij*, Elisa M. Tjon Kon Fat▾, Susan J.F. van den Eeden*, Louis Wilson*, Moises Batista da Silva†, Claudio G. Salgado†, John S. Spencer¶, Paul L.A.M. Corstjens▾, and Annemieke Geluk*

**Dept. of Infectious Diseases, ▾Dept. Molecular Cell Biology and Dept. Reumatology, Leiden University Medical Center, The Netherlands and ¶ Dept. of Microbiology, Immunology and Pathology, Colorado State University, Fort Collins, USA. †Laboratório de Dermato-Imunologia, Instituto de Ciências Biológicas, Universidade Federal do Pará, Marituba, Pará, Brazil.*

RUNNING TITLE: User-friendly test for anti-PGL-I detection

KEYWORDS: immune-monitoring, lateral flow assay, leprosy, PGL-I, serology, user-friendly, rapid test

CORRESPONDENCE TO:

Anouk van Hooij, Dept. of Infectious Diseases, LUMC

PO Box 9600, 2300 RC Leiden, The Netherlands

Tel: +31-71-526-3844; E-mail: A.van_Hooij@lumc.nl

Supplementary Figure S1: Heatmap of ELISA, UCP-LFA and Gold-LFA test results per cohort.

Heatmap showing the positive test results (green) for ELISA (OD450-background >0.200), UCP-LFA (ratio >0.29) and Gold-LFA (visual score >0.5) for the Philippine, Bangladeshi and Brazilian cohort. The bacterial index (BI) is shown if assessed (BI+ = green; BI- = orange). Values range from 0-2,605 for ELISA, 0-31 for the UCP-LFA and from 0-4 for the Gold -LFA. MB = multibacillary, PB = paucibacillary, NEC = non-endemic controls, HHC = healthy household contacts, HHC&BCG = BCG-vaccinated healthy household contacts, EC = endemic controls.

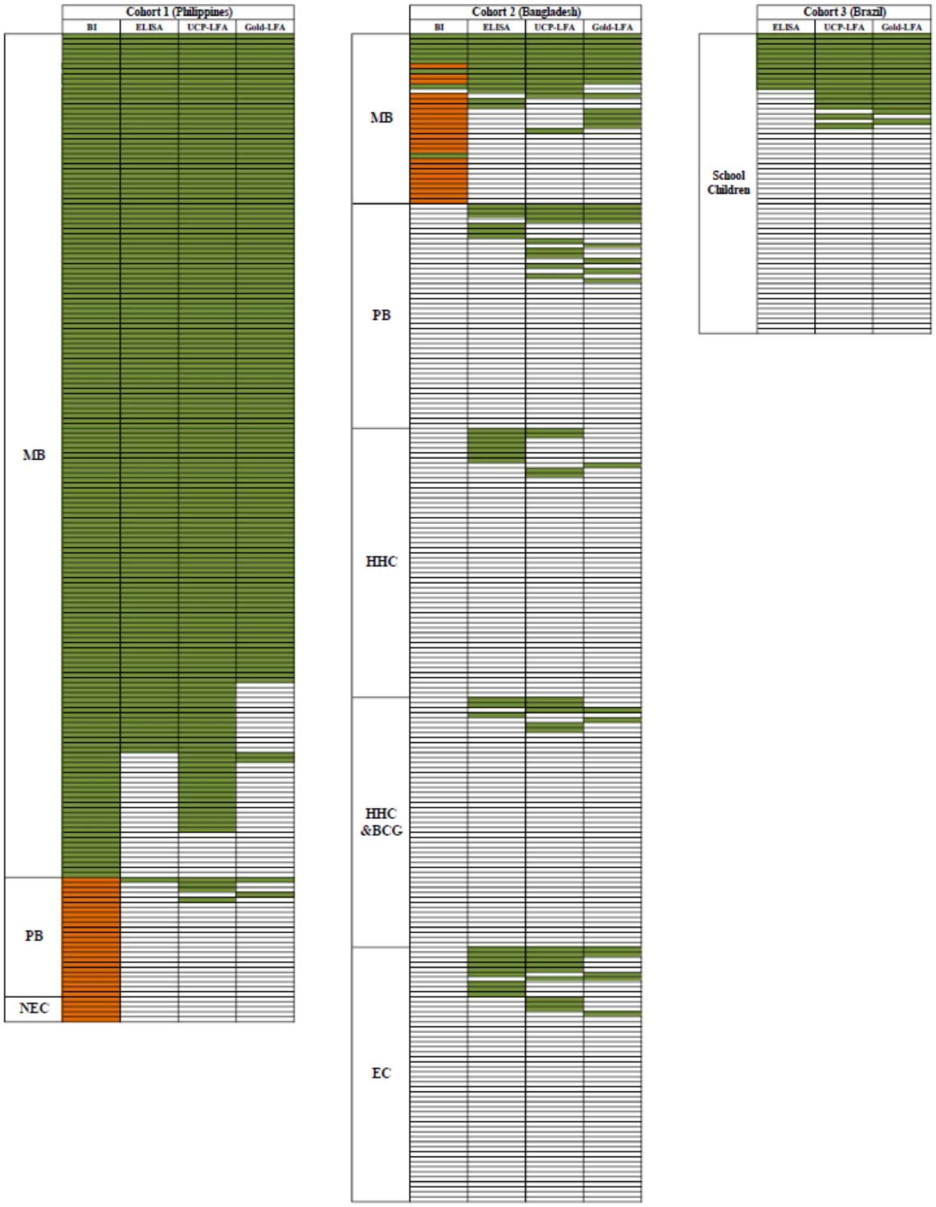
Cohort 1 (Philippines)
BI
ELISA
UCP-LFA
Gold-LFA
MB
PB
NEC
Cohort 2 (Bangladesh)
BI
ELISA
UCP-LFA
Gold-LFA
MB
PB
HHC
HHC &BCG
EC
Cohort 3 (Brazil)
ELISA
UCP-LFA
Gold-LFA
School Children

Supplementary Figure S2: Heatmap of UCP-LFA and Gold-LFA nonconcordant test results in leprosy patients.

Heatmap showing test results of leprosy patients (MB and PB) from the Philippine and Bangladeshi cohort with a positive result in either the UCP-LFA or Gold-LFA. Positive test results were divided into two groups, weak positives (light green; UCP-LFA : 0,29-0,4 ; Gold-LFA : 0,5) and strong positives (dark green; UCP-LFA ≥ 0,4; Gold-LFA ≥1). MB = multibacillary, PB = paucibacillary.

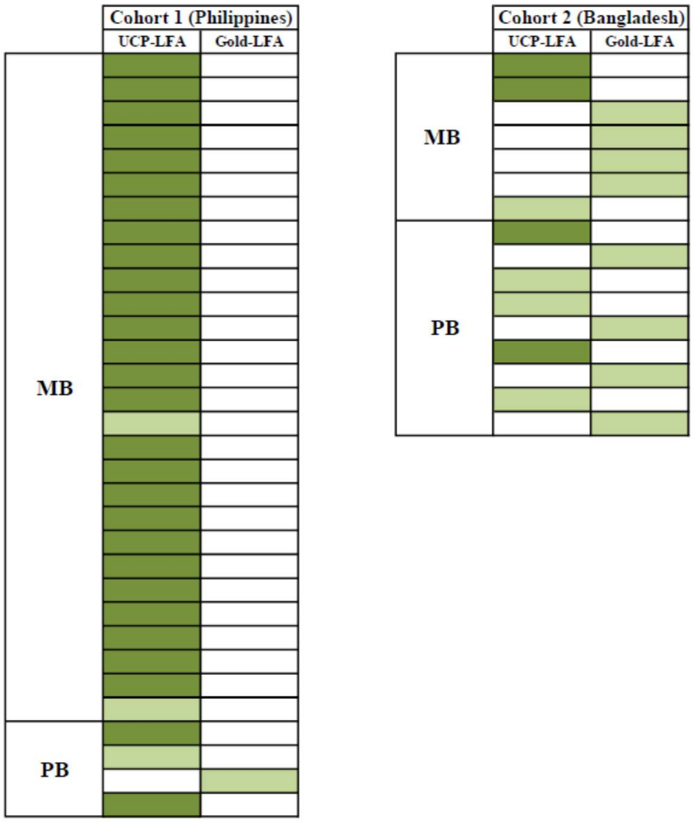
Cohort 1 (Philippines)
UCP-LFA
Gold-LFA
MB
PB
Cohort 2 (Bangladesh)
UCP-LFA
Gold-LFA
MB
PB

	ELISA	UCP-LFA	Gold-LFA
TB1	0,024	0,09	0
TB2	0,05	0,06	0
TB3	0,055	0,24	0
TB4	0,042	0,00	0
TB5	0,082	0,11	0
TB6	0,046	0,10	0
TB7	0,071	0,11	0
TB8	0,061	0,24	0
TB9	0,055	0,20	0
TB10	0,024	0,04	0
TB11	0,025	0,11	0
TB12	0,009	0,03	0
TB13	0,005	0,10	0
TB14	0,022	0,14	0
TB15	0,09	0,16	0
TB16	0,004	0,01	0
TB17	0,033	0,13	0
TB18	0,034	0,08	0

Supplementary Table S1: Evaluation of ELISA, UCP-LFA and Gold-LFA in patients with active tuberculosis.

Test results for 18 patients with active tuberculosis in PGL-I ELISA (threshold for positivity: OD_{450}-background >0.200), UCP-LFA (threshold for positivity :ratio >0.29) and Gold-LFA (threshold for positivity: visual score >0.5).
